# Correlation between serum carnosinase concentration and renal damage in diabetic nephropathy patients

**DOI:** 10.1007/s00726-021-02975-z

**Published:** 2021-04-03

**Authors:** Zhou Zhou, Xue-qi Liu, Shi-qi Zhang, Xiang-ming Qi, Qiu Zhang, Benito Yard, Yong-gui Wu

**Affiliations:** 1 Department of Nephropathy, the First Affiliated Hospital of Anhui Medical University, Hefei, 230022 Anhui People’s Republic of China; 2 Center for Scientific Research of Anhui Medical University, Hefei, 230022 Anhui People’s Republic of China; 3 Department of Endocrinology, the First Affiliated Hospital of Anhui Medical University, Hefei, 230022 Anhui People’s Republic of China; 4 Vth Department of Medicine (Nephrology/Endocrinology/Rheumatology) University Medical Center Mannheim, University of Heidelberg, Theodor-Kutzer-Ufer1-3, 68167 Mannheim, Germany

**Keywords:** Diabetic nephropathy, Carnosinase, Injury, Albuminuria

## Abstract

Diabetic nephropathy (DN) is one of the major complications of diabetes and contributes significantly towards end-stage renal disease. Previous studies have identified the gene encoding carnosinase (CN-1) as a predisposing factor for DN. Despite this fact, the relationship of the level of serum CN-1 and the progression of DN remains uninvestigated. Thus, the proposed study focused on clarifying the relationship among serum CN-1, indicators of renal function and tissue injury, and the progression of DN. A total of 14 patients with minimal changes disease (MCD) and 37 patients with DN were enrolled in the study. Additionally, 20 healthy volunteers were recruited as control. Further, DN patients were classified according to urinary albumin excretion rate into two groups: DN with microalbuminuria (*n* = 11) and DN with macroalbuminuria (*n* = 26). Clinical indicators including urinary protein components, serum carnosine concentration, serum CN-1 concentration and activity, and renal biopsy tissue injury indexes were included for analyzation. The serum CN-1 concentration and activity were observed to be the highest, but the serum carnosine concentration was the lowest in DN macroalbuminuria group. Moreover, within DN group, the concentration of serum CN-1 was positively correlated with uric acid (UA, *r* = 0.376, *p* = 0.026) and serum creatinine (SCr, *r* = 0.399, *p* = 0.018) and negatively correlated with serum albumin (Alb, *r* = − 0.348, *p* = 0.041) and estimated glomerular filtration rate (eGRF, *r* = − 0.432, *p* = 0.010). Furthermore, the concentration of serum CN-1 was discovered to be positively correlated with indicators including 24-h urinary protein–creatinine ratio (24 h-U-PRO/CRE, *r* = 0.528, *p* = 0.001), urinary albumin-to-creatinine ratio (Alb/CRE, *r* = 0.671, *p* = 0.000), urinary transferrin (TRF, *r* = 0.658, *p* = 0.000), retinol-binding protein (RBP, *r* = 0.523, *p* = 0.001), N-acetyl-glycosaminidase (NAG, *r* = 0.381, *p* = 0.024), immunoglobulin G (IgG, *r* = 0.522, *p* = 0.001), cystatin C (Cys-C, *r* = 0.539, *p* = 0.001), beta-2-microglobulin (β2-MG, *r* = 0.437, *p* = 0.009), and alpha-1-macroglobulin (α1-MG, *r* = 0.480, *p* = 0.004). Besides, in DN with macroalbuminuria group, serum CN-1 also showed a positive correlation with indicators of fibrosis, oxidative stress, and renal tubular injury. Taken together, our data suggested that the level of CN-1 was increased as clinical DN progressed. Thus, the level of serum CN-1 might be an important character during the occurrence and progression of DN. Our study will contribute significantly to future studies focused on dissecting the underlying mechanism of DN.

## Introduction

Diabetes mellitus is a group of metabolic diseases characterized by chronic hyperglycemia caused by various factors. As reported, the morbidity and mortality rates of diabetes are increasing annually. Long-term metabolism disorders could lead to several chronic and progressive diseases in multiple tissues and organs, and further cause functional impairment even failure. Many studies have focused on prevention and treatment of complications of diabetes mellitus effectively. Diabetic nephropathy (DN), as one of the major chronic complications of diabetes, has become a major cause of end-stage renal disease (ESRD). The complicated pathogenesis of DN has damped the current treatment of DN. Related studies have shown that the occurrence and progression of DN are related to several factors, including hemodynamic disorder, glycolipid metabolism disorder, accumulation of advanced glycosylation products (AGEs) and advanced lipid products (ALEs), activation of renin–angiotensin–aldosterone system (RAAS), oxidative stress response, overexpression of various vasoactive substances and cytokines, heredity and so on (Cheng et al. 2019; Guariguata et al. 2014; Whyte et al. 2019). DN is characterized by progressive kidney damage, mainly manifested in increased urinary albumin and renal function damage. Furthermore, albuminuria has been used as indicator of DN at early stage for diagnosis (Perkins et al. 2007; Koye et al. 2018). However, in many cases, the appearance of microalbuminuria and renal impairment do not happen simultaneously (Krolewski 2015; Robles et al. 2015). In some countries, such as China, patients are well-aware the change of renal function with low attention in microalbuminuria. Patients with DN will be benefited if diagnosed and treated at early stage.

Carnosine, a water-soluble dipeptide, is consist of β-alanine and l-histidine. As a natural dipeptide, carnosine is widely distributed in various organs of human body. Carnosine plays an important role in various biological activities including balance physiological pH, chelate metal ions, antioxidant stress, inhibit inflammatory factor expression, inhibit RAAS activity, inhibit AGEs and ALEs (Boldyrev et al. 2013). Based on these facts, carnosine has been applied to treat the adverse effects of high glucose levels on renal cells (Janssen et al. 2005). Previous studies suggested that carnosine could improve diabetic nephropathy by inhibiting the formation of advanced glycosylation and advanced lipoylation products and further suppressing carbonyl stress and inflammation (Lee et al. 2005; Aldini et al. 2011; Menini et al. 2012). During the metabolic process, carnosine is mainly synthesized by carnosine synthase and degraded by carnosinase. There are two kinds of carnosinases in vivo including: serum carnosinase (CN-1) and cytosolic nonspecific dipeptidase (CN-2). CN-1 is specifically expressed in brain, blood, kidney, skeletal muscle and etc. Meanwhile, CN-2 is widely distributed in human tissues, but not in serum and cerebrospinal fluid. Their corresponding carnosinases are *CNDP1* and *CNDP2*, which are localized side by side on human chromosome 18 in a head-to-tail position (Lenney et al. 1982; Pegova et al. 2000; Peters et al. 2010). Studies have found that polymorphisms in *CTG* repeats in signal peptide coding regions are associated with susceptibility to DN in *CNDP1* genes encoding the peptide. In addition, several independent clinical studies (Freedman et al. 2007; Chakkera et al. 2011) and biological data (Sauerhöfer et al. 2007) have confirmed the relationship between *CNDP1* and DN. As reported, *CTG* polymorphisms could affect CN-1 secretion. When *CTG* repeats are short (such as homozygous *(CTG)5*), the number of hydrophobic leucine residues on the encoded CN-1 signal peptide is small. In this case, the newly synthesized CN-1 is more likely to remain in cells without being secreted into the blood. As a result, the low concentrations of serum CN-1 will function as antioxidant and anti-glycosylated in response to the relative increase of carnosine levels (Riedl et al. 2007).

However, the exact role of carnosine/CN-1 system in DN remains uninvestigated clinically. Taken from previous studies, we proposed that the greatest effect of serum CN-1 levels on the availability of carnosine is due to the ability of this dipeptidase to regulate carnosine availability. To verify the hypothesis, the present study examined the expression of CN-1 in renal tissues at different stages in patients with type 2 diabetic nephropathy. Further, we also measured the serum expression of CN-1 and analyzed the correlation of CN-1 expression with relevant clinical indicators in DN patients. From there, we also examined the relationship between the expression levels of CN-1 and progression of renal impairment in diabetic patients.

## Methods

### Patients

A total of 14 minimal changes disease (MCD) patients and 37 DN patients were enrolled in the study. All patients were recruited in the time frame from January 2017 to December 2019 from the Department of nephrology of First Affiliated Hospital of Anhui Medical University. At the same time, the fasting serum of 20 normal subjects in the physical examination center of our hospital was collected as control. This study was approved by the Ethics Committee of the First Affiliated Hospital of Anhui Medical University (Hefei, China, approval no.: PJ2018-05-09). All participants signed written informed consent prior to participating in the study.

DN inclusion criteria: (1) diagnosis of diabetes mellitus, which included fasting plasma glucose ≥ 126 mg/dL (7.0 mmol/L). Fasting was defined as no caloric intake for at least 8 h, or 2 h postprandial blood glucose ≥ 200 mg/dL (11.1 mmol/L) during an oral glucose tolerance test (American Diabetes Association 2013); (2) the pathological diagnosis of renal biopsy is in accordance with the diagnostic criteria of diabetic nephropathy. Exclusion criteria were: (1) type 1 diabetes mellitus; (2) suffering from other diseases or complications, such as primary nephropathy and other secondary nephropathies, heart failure, malignant hypertension, myocardial infarction, cerebrovascular accident, infect, liver dysfunction with alanine aminotransferase level exceedingly twice the upper normal limit; (3) pregnant and lactating women; (4) cancer; and (5) those lost for other reasons. According to the magnitude of albuminuria, patients were categorized into 2 subgroups: microalbuminuria (urinary albumin excretion 30–300 mg/24 h) and macroalbuminuria (urinary albumin excretion > 300 mg/24 h).

The control group included patients with normal blood glucose (fasting plasma glucose < 6.1 mmol/L, or 2 h postprandial blood glucose < 7.8 mmol/L during an oral glucose tolerance test) and MCD by renal biopsy as the renal tissue of MCD patients had no obvious pathological changes under light microscope. Exclusion criteria were: (1) hypertension; (2) suffering from other diseases or complications, such as secondary nephropathies, heart failure, malignant hypertension, myocardial infarction, cerebrovascular accident, infect, liver dysfunction with alanine aminotransferase level exceedingly twice the upper normal limit; (3) pregnant and lactating women; (4) cancer; and (5) those lost for other reasons.

### Renal histology analysis

After obtaining the patient’s consent and excluding contraindications, we have obtained renal tissue from ultrasound-guided renal biopsy under local anesthesia. The renal histopathological changes were observed by PAS and MASSON staining. The important indicators, such as the glomerular volume, mesangial cells proliferate and matrix expands, tubular epithelial cell atrophy and lumen dilatation, were measured and recorded for further analyzation. Each biopsy contains at least 8 glomeruli for evaluation. In brief, Tervaert classification was used to evaluate glomerular lesions. Class I: glomerular basement membrane thickening. Class II: mesangial expansion, mild (IIa) or severe (IIb). Class III: nodular sclerosis (Kimmelstiel–Wilson lesions). Class IV: advanced diabetic glomerulosclerosis. Interstitial fibrosis and tubular atrophy (IFTA) were scored as follows: 0, absent; 1, < 25%; 2, 25–50% and 3, > 50% of the total area. Interstitial inflammation was scored as follows: 0, absent; 1, inflammation only in relation to IFTA and 2, inflammation in areas without IFTA. Arteriolar hyalinosis was scored as follows: 0, absent; 1, at least one area of arteriolar hyalinosis and 2, more than one area of arteriolar hyalinosis. Arteriosclerosis was scored as follows: NA, absent of large vessels; 0, no intimal thickening; 1, intimal thickening less than thickness of media and 2, intimal thickening greater than thickness of media (An et al. 2015). The glomerular and tubulointerstitial lesions of ten visual fields were randomly captured by Image J software under microscope. The scores of mesangial expansional index and tubulointerstitial injury index of renal tissue were obtained.

### Renal immunohistochemistry

Paraffin-embedded renal tissue was continuously sliced and collected on tissue adhesion microscope slides. After deparaffinization and rehydration, tissue sections were washed, and then incubated with peroxidase blockers for 30 min at 37 °C. Next, the antigen was repaired, and the slides were put into boiling sodium citrate and heated for 4 min. After cooling to room temperature, tissue slices were blocked in goat serum at 37 °C for 30 min to prevent background staining. Subsequently, tissue sections were incubated with anti-carnosine dipeptidase 1 (1:300, Sigma, California, USA, HPA008933), anti-fibronectin (1:200, Abcam, Cambridge, UK, ab2413), anti-collagen IV (1:200, Proteintech, Wuhan, China, 55131-1-AP), anti-collagen I (1:200, Abcam, Cambridge, UK, ab34710), anti-8-hydroxy-2′-deoxyguanosine (1:200, Abcam, Cambridge, UK, ab48508), anti-4-hydroxynonenal (1:200, Abcam, Cambridge, UK, ab46545), anti-tumor necrosis factor-α (1:200, Affinity, OH, USA, AF7014), anti-monocyte chemoattractant protein-1 (1:400, Arigobio, Taiwan, China, ARG57649), and anti-interleukin-1β (1:200, Affinity, OH, USA, DF6251) and anti-kidney injury molecule-1 (1:200, Abcam, Cambridge, UK, ab78494) overnight at 4 °C. After wash, the tissue sections were incubated with a secondary antibody for 40 min at 37 °C. Then, 3,3′ diaminobenzidine (DAB) was added chromogenic for visualization in a color reaction, followed by hematoxylin dye. Brown staining represented positive results. All images were taken with a Zeiss microscopy and the intensity of the immunostaining was quantified using Image J analysis software.

### Serum carnosine concentrations measurements

Serum carnosine concentrations measured by a UPLC Dionex Ultimate 3000 chromatographic system (Thermo Scientific, San Jose, USA) consisting of binary pump, degasser, autosampler and column oven coupled to a Q-Exactive plus hybrid quadrupole-orbitrap mass spectrometer (Thermo Scientific, San Jose, USA) with a heat electrospray ionization (HESI) through Targeted selected ion monitoring (T-SIM) mode. The carnosine stock solution was dissolved in water, the calibration lines were prepared at seven spiking levels (0.5, 1, 5, 10, 20, 50 and 100 ng/mL) by diluting stock solution at ratio of acetonitrile: water = 7:3. For serum samples, an aliquot of 60 μL serum was pipetted into centrifuge tube, 150 μL cold acetonitrile (− 20 °C) was added for protein precipitation, after vortexing for 30 s, the sample proceeded with high-speed centrifugation (12,000×*g* for 20 min at 4 °C). The separation was performed in a Waters Acquity UPLC BEH Amide column (100 mm × 2.1 mm, 1.7 µm, Waters, USA). Isocratic mobile solvents consisted of acetonitrile: water (containing 0.1% formic acid) = 7:3, The flow rate was 0.3 mL/min, and the injection volume was 5 µL, the column temperature was maintained at 35 °C. The conditions used for mass spectrometer were as follows: spray voltage: + 4.0 kV; capillary temperature: 320 °C; auxiliary gas heater temperature: 100 °C; S-lens RF level: 50 V; by T-SIM mode, the inclusion list was set with C9H14O3N4; resolution: 17,500; Automatic gain control (AGC) target: 5.0e^4^; loop count: 1; Maximum injection time (IT): 100 ms; isolation window: 4.0 *m*/*z*; Q-Exactive 2.9 (Thermo Fisher Scientific, San Jose, USA), Xcalibur 4.1 software (Thermo Fisher Scientific, San Jose, USA) was used for data acquisition and analysis.

### Serum CN-1 concentrations and activity measurements

5 mL of venous blood was obtained from each patient and serum was extracted for experiments. Serum CN-1 concentrations were measured using enzyme-linked immunosorbent assay (ELISA) kits (R&D Systems), according to the manufacturer’s instructions. Serum carnosinase activity was determined according to the method described by Teufel et al. (2003). In brief, Carnosinase activity in human plasma was determined with 5 μL sample. The reaction was initiated by adding 50ul of 1% trichloroacetic acid. Liberated histidine was derivatized by adding 50 μL of 5 mg/mL *o*-pthaldialdehyde dissolved in 2 M NaOH and 30 min of incubation at 30 °C. Fluorescent signals were captured using multifunctional enzyme marker reader.

### Statistical analysis

All statistical analyses were carried out with version 22 (IBM Corporation, Armonk, NY). The normality of the data was analyzed by the Shapiro–Wilk test and quantile–quantile plots. Qualitative indicators (counting data) were compared across three groups using the chi-square test. Quantitative indicators (measurement data) were represented as mean ± standard deviation, consistent with the normal distribution of the inter-group mean comparison single factor analysis of variance (ANOVA) followed by a post hoc Bonferroni test for multiple comparisons. For non-normally distributed data [denoted by median (p25, p75)], we used Kruskal–Wallis ANOVA to evaluate differences among groups followed by a Dunn–Bonferroni test for post hoc comparisons. The linear correlation was analyzed by Pearson test or Spearman test. *p* < 0.05 was considered statistically significant in all analyses.

## Results

### Patient characteristics

A total of 51 patients criteria were divided into three groups as described earlier: MCD group (*n* = 14), DN with microalbuminuria group (*n* = 11), and DN with macroalbuminuria group (*n* = 26). Demographic, clinical, and laboratory data of patients were listed in Table 1. In MCD group, there were 8 males and 6 females with an average age of 40.86 ± 13.34 years. In DN with microalbuminuria group, there were 7 males and 4 females with an average age of 52.18 ± 5.81 years. And in DN with macroalbuminuria group, there were 19 males and 7 females with an average age of 50.08 ± 7.17 years. There was no significant difference observed among three groups in age, sex, body mass index (BMI), diastolic blood pressure (DBP), serum uric acid (UA) and C-reactive protein (CRP) (*p* > 0.05). Other indicators, however, including systolic blood pressure (SBP) (*p* = 0.000), fasting blood glucose (FBG) (*p* = 0.015), glycosylated hemoglobin (HbA1c) (*p* = 0.000), albumin (Alb) (*p* = 0.000), blood urea nitrogen (BUN) (*p* = 0.000), serum creatinine (SCr) (*p* = 0.000), estimated glomerular filtration rate (eGRF) (*p* = 0.000), total cholesterol (TC) (*p* = 0.000), triglyceride (TG) (*p* = 0.049), Urinary albumin excretion rate (U-AER) (*p* = 0.000), 24-h urinary protein creatinine ratio (24 h-U-PRO/CRE) (*p* = 0.000), were significantly different among three groups.

**Table 1:** Baseline demographic, clinical, and laboratory data of patients

	MCD (*n* = 14)	DN		*F*/*χ*^2^	*p* value
		Microalbuminuria (*n* = 11)	Macroalbuminuria (*n* = 26)		
Age (years)	40.86 ± 13.34	52.18 ± 5.81	50.08 ± 7.17	6.213^a,b^	0.004
Sex (male/female)*	8/6	7/4	19/7	1.098	0.578
BMI (kg/m^2^)	24.69 ± 2.35	24.63 ± 2.00	25.52 ± 3.07	0.646	0.528
SBP (mmHg)	120.93 ± 9.75	134.09 ± 18.10	141.62 ± 15.14	9.124^a,b^	0.000
DBP (mmHg)	79.36 ± 6.78	83.36 ± 13.60	84.62 ± 10.67	1.154	0.324
FBG (mmol/L)	4.94 ± 0.47	7.68 ± 3.16	7.88 ± 3.69	4.593^a,b^	0.015
HbA1c (%)	4.92 ± 0.35	7.87 ± 1.54	7.46 ± 1.63	19.187^a,b^	0.000
Alb (g/L)	20.85 ± 5.55	41.4 ± 2.36	34.76 ± 7.44	37.888^a,b,c^	0.000
BUN (mmol/L)	5.00 ± 1.63	6.5 ± 2.17	8.89 ± 3.5	9.107^b^	0.000
UA (μmol/L)	382.07 ± 134.07	372.64 ± 119.03	399.92 ± 72.42	0.318	0.729
SCr (μmol/L)	71.09 ± 24.54	76.72 ± 34.23	125.51 ± 53.67	9.065^b^	0.000
eGFR (mL/min/1.73m2)	110.14 ± 27.36	99.18 ± 25.76	68.00 ± 30.38	11.250^a,b^	0.000
TC (mmol/L)	8.36 ± 2.04	3.78 ± 0.38	5.26 ± 1.5	30.948^a,b,c^	0.000
TG (mmol/L)	1.84 ± 0.58	2.54 ± 0.80	1.8 ± 0.96	3.218	0.049
CRP (mg/L)	0.89 ± 0.69	1.48 ± 1.21	1.45 ± 0.82	2.093	0.134
U-AER (mg/24 h)	5490.71 ± 2872.62	164.21 ± 103.18	2534.31 ± 2215.21	18.731^a,b,c^	0.000
24 h-U-PRO/CRE	6.2 ± 3.49	0.41 ± 0.3	4.99 ± 4.00	9.817^a,b^	0.000

Data are expressed as mean ± SD, or number

*BMI* body mass index, *SBP* systolic blood pressure, *DBP* diastolic blood pressure, *FBG* fasting blood glucose, *HbA1c* glycosylated hemoglobin, *Alb* albumin, *BUN* blood urea nitrogen, *UA* uric acid, *SCr* serum creatinine, *eGFR* estimated glomerular filtration rate, *TC* total cholesterol, *TG* triglyceride, *CRP* C-reactive protein, *U-AER* Urinary albumin excretion rate, *24 h-U-PRO/CRE* 24-h urinary protein creatinine ratio

*Chi-square inspection

^a^*p* < 0.05 (post hoc), DN with microalbuminuria vs. MCD

^b^*p* < 0.05 (post hoc), DN with macroalbuminuria vs. MCD

^c^*p* < 0.05 (post hoc), DN with microalbuminuria vs. DN with macroalbuminuria

The components of urinary protein from three groups were summarized in Table 2. Significant differences were observed in components including transferrin (TRF) (*p* = 0.000), retinol-binding protein (RBP) (*p* = 0.000), *N*-acetyl-glycosaminidase (NAG) (*p* = 0.000), IgG, fibrin degradation product (FDP) (*p* = 0.019), cystatin C (Cys-C) (*p* = 0.001), beta-2-microglobulin (β2-MG) (*p* = 0.007), alpha-1-macroglobulin (α1-MG) (*p* = 0.004), albumin-to-creatinine (Alb/CRE) (*p* = 0.000). Compared with the DN group, the TRF, NAG, IgG and Alb/CRE levels of the MCD group were significantly higher. Within both DN groups, all components of urinary protein were significantly higher in DN with macroalbuminuria group than those in DN with microalbuminuria group.

**Table 2:** Urinary protein composition of patients

	MCD (*n* = 14)	DN		*χ*^2^	*p* value
		Microalbuminuria (*n* = 11)	Macroalbuminuria (*n* = 26)		
TRF (mg/L)	148.56 (137.13, 239.46)	6.82 ± 4.59	71.66 (38.42, 118.87)	30.692^a,b,c^	0.000
RBP (mg/L)	0.13 (0.08, 0.43)	0.15 (0.07, 0.55)	3.1 (0.33, 8)	17.998^a,b,c^	0.000
NAG (U/L)	24.46 (19.43, 45.03)	8.59 ± 4.46	16.16 (9.85, 29.93)	16.128^a,b,c^	0.000
IgG (mg/L)	155.11 (82.96, 355.63)	4.02 (1.57, 18)	100.94 (51.52, 237.32)	23.974^a,b,c^	0.000
FDP (μg/L)	0.05 (0.02, 0.1)	0.06 (0.03, 0.1)	0.1 (0.06, 0.54)	7.889^a,b,c^	0.019
Cys-C (mg/L)	0.08 ± 0.09	0.1 (0.03, 0.13)	0.41 (0.13, 1.39)	14.953^a,b,c^	0.001
β2-MG (mg/L)	0.15 (0.08, 0.3)	0.08 ± 0.05	0.65 (0.18, 3.67)	10.066^a,b,c^	0.007
α1-MG (mg/L)	23.37 (14.69, 53.89)	0.29 ± 0.26	28.7 (10.76, 58.73)	11.048^a,b,c^	0.004
Alb/CRE (mg/gcr)	3574.96 ± 1663.69	157.27 ± 142.56	1647.68 (870.30, 3944.87)	27.288^a,b,c^	0.000

Data are expressed as mean ± SD, median (interquartile range), or number

*TRF* transferrin, *RBP* retinol-binding protein, *NAG* *N*-acetyl-glycosaminidase, *IgG* Immunoglobulin G, *FDP* fibrin degradation product, *Cys-C* cystatin C, *β2-MG* beta-2-microglobulin, *α1-MG* alpha-1-macroglobulin, *Alb/CRE* albumin-to-creatinine ratio

^a^*p* < 0.05 (post hoc), DN with microalbuminuria vs. MCD

^b^*p* < 0.05 (post hoc), DN with macroalbuminuria vs. MCD

^c^*p* < 0.05 (post hoc), DN with microalbuminuria vs. DN with macroalbuminuria

### Serum carnosine and CN-1 concentration

**Fig. 1:**
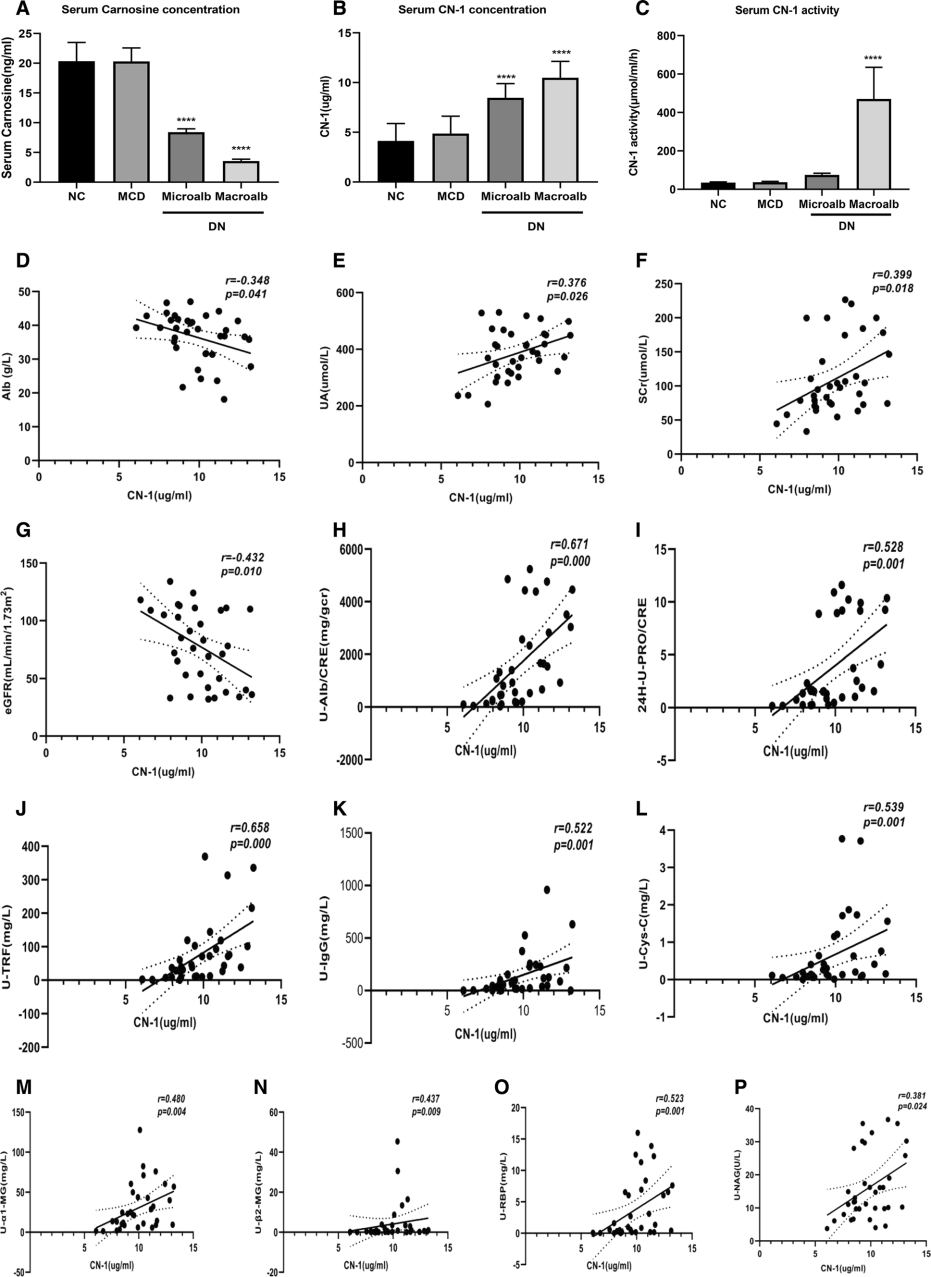
Serum carnosine and CN-1 concentration, and its relationship with clinical indicators. The serum carnosine concentration was detected by UPLC (**a**). The serum CN-1 concentration and activity were detected by ELISA method (**b**, **c**). Correlations between serum CN-1 concentration with Alb (**d**), UA (**e**), SCr (**f**), eGRF (**g**), U-Alb/CRE (**h**), 24 h-U-PRO/CRE (i), U-TRF (**j**), U-IgG (**k**), U-Cys-C (**l**), U- α1-MG (**m**), U-β2-MG (**n**), U-NAG (**o**), U-RBP (**p**) in DN group. *TRF* transferrin, *RBP* retinol-binding protein, *NAG* *N*-acetyl-glycosaminidase, *IgG* Immunoglobulin G, *FDP* fibrin degradation product, *Cys-C* cystatin C, *β2-MG* beta-2-microglobulin, *α1-MG* alpha-1-macroglobulin, *Alb/CRE* albumin-to-creatinine ratio. *p < 0.05; **p < 0.01; ***p < 0.001; ****p < 0.0001

To understand the metabolism of carnosine in human body, we used UPLC-MS/MS method to detect the content of serum carnosine in each group. We found that the content of human serum carnosine was low. Compared with the normal control group, there was no significant difference in the level of serum carnosine in MCD group, while the content of serum carnosine in DN group was significantly reduced. With the occurrence and development of DN, the content of serum carnosine gradually decreased. There was statistical significance (Fig. 1a.). With analyzation of serum carnosine and CN-1 concentrations in all groups, the serum CN-1 concentration was the highest in DN with macroalbuminuria group, followed by microalbuminuria group, and the lowest in MCD group (Fig. 1b.). In accordance with the behavior of serum CN-1 concentration, serum CN-1 activity was the highest in DN with macroalbuminuria group and lowest in MCD group (Fig. 1c).

### Serum CN-1 concentration relationship with clinical indicators

In DN groups, examining with other clinical indicators, serum CN-1 concentration was positively correlated with UA (*r* = 0.376, *p* = 0.026) and SCr (*r* = 0.399, 0.018). In contrast, it was negatively correlated with serum Alb (*r* = − 0.348, *p* = 0.041) and eGRF (*r* = − 0.432, *p* = 0.010) (Fig. 1d–g). Taken other factors into consideration, there was no correlation found between serum CN-1 concentration and age, sex, BMI, blood pressure, FBG, HbA1c, blood lipids and CRP. In addition, serum CN-1 concentration positively correlated with 24 h-U-PRO/CRE (*r* = 0.528, *p* = 0.001), urinary Alb/CRE (*r* = 0.671, *p* = 0.000), urinary TRF (*r* = 0.658, *p* = 0.000), RBP (*r* = 0.523, *p* = 0.001), NAG (*r* = 0.381, *p* = 0.024), IgG (*r* = 0.522, *p* = 0.001), Cys-C (*r* = 0.539, *p* = 0.001), β2-MG (*r* = 0.437, *p* = 0.009), α1-MG (*r* = 0.480, *p* = 0.004) (Fig. 1h–p). Further, there is no significant correlation with FDP.

### DN patients show more severe pathological changes and highly expression of CN-1 in the kidney.

**Fig. 2:**
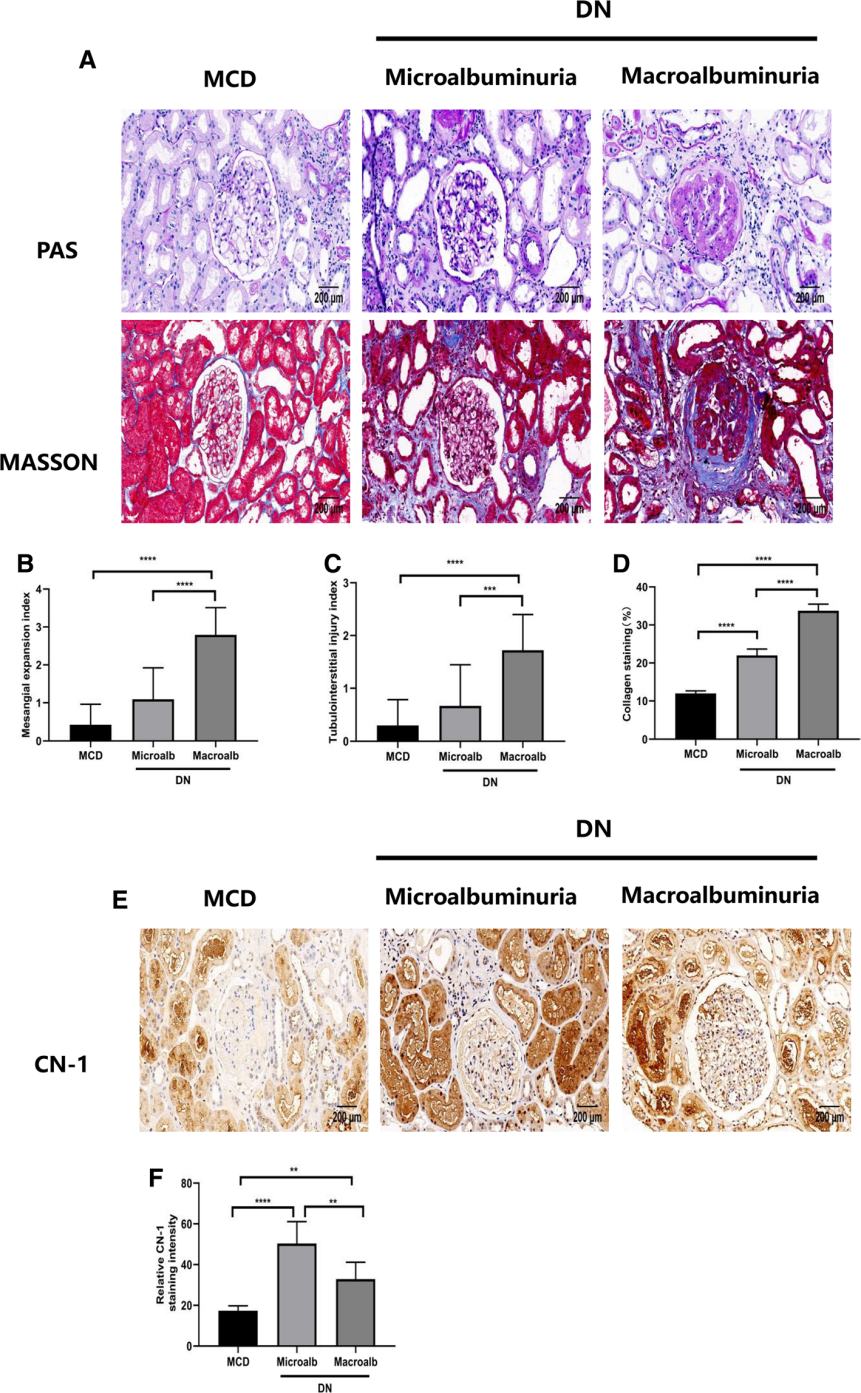
Expression of CN-1 was increased in renal biopsies of DN patients. PAS, and Masson’s staining of the indicated human renal biopsies (**a**). Quantitative analysis of PAS staining (**b**, **c**) and Masson’s trichrome staining (**d**) of the MCD and DN individuals. Immunohistochemical (IHC) staining of CN-1 in three groups (**e**). Quantitative results of CN-1 in IHC staining (**f**). Representative microscopic images are shown (× 200 magnification). *p < 0.05; **p < 0.01; ***p < 0.001; ****p < 0.0001

Compared with MCD group, glomerular mesangial expansion and tubulointerstitial injury were further aggravated with the increase of proteinuria in DN (Fig. 2a–d). We evaluated the kidney damage in the DN group, as shown in Table 3. To test whether CN-1 expression corresponded with pathological changes, we examined CN-1 expression in kidney by immunohistochemistry and observed that CN-1 was mainly expressed in renal tubules. (Fig. 2e, f). Collected data suggested that the expression level of CN-1 was significantly higher in patients with DN than MCD. Further, the CN-1 concentration was lower in macroalbuminuria DN group than microalbuminuria DN group.

**Table 3:** Pathologic features of diabetic nephropathy patients

	Microalbuminuria (*n* = 11)	Macroalbuminuria (*n* = 26)		*p* value
Glomerular lesions				
Class I	2	0	21.417	0.000
Class IIa	7	2		
Class IIb	1	3		
Class III	1	19		
Class IV	0	2		
IFTA				
0	5	0	13.143	0.004
1	4	11		
2	2	13		
3	0	2		
Interstitial inflammation				
0	5	1	10.164	0.006
1	4	13		
2	2	12		
Arteriolar hyalinosis				
0	4	0	11.409	0.003
1	6	12		
2	1	14		
Arteriosclerosis				
0	7	1	16.514	0.001
1	3	18		
2	1	7		

Data are expressed as number

*IFTA* interstitial fibrosis and tubular atrophy

### The serum CN-1 expression was associated not only with renal fibrosis but also with tubule damage in DN patients

**Fig. 3:**
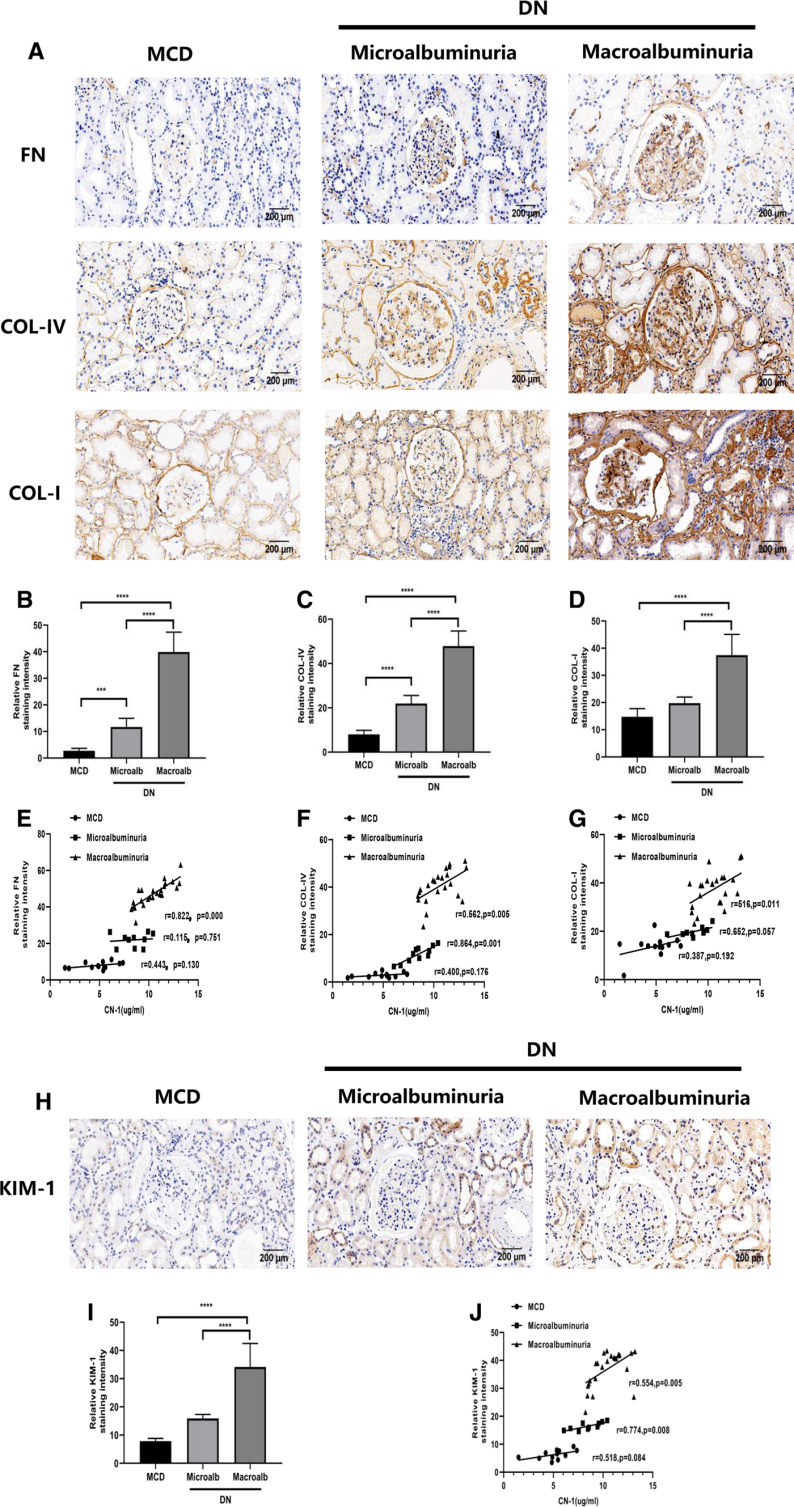
Renal fibrosis and injury correlate with serum CN-1 overexpression in DN with macroalbuminuria. IHC staining of fibrosis indexes in three groups, including FN, COL-IV, COL-I (**a**). Quantitative results of FN (**b**), COL-IV (**c**), COL-I (**d**) in IHC staining. Correlation of serum CN-1 expression with various parameters, including FN (**e**), COL-IV (**f**), COL-I (**g**). IHC staining of oxidative stress indexes KIM-1 (**h**) in three groups. Quantitative results of KIM-1 (**i**) in IHC staining. Correlation of serum CN-1 expression with KIM-1 (**j**). Representative microscopic images are shown (× 200 magnification). *p < 0.05; **p < 0.01; ***p < 0.001; ****p < 0.0001

To understand the role of CN-1 in kidney fibrosis, we examined the renal tissue expressions of fibronectin (FN), collagen IV (COL-IV) and collagen I (COL-I) in each group (Fig. 3a–d). With the increase of proteinuria in DN patients, the indicators of fibrosis in kidney further aggravated. The correlations between expressions of FN, Col-IV, Col-I and serum CN-1 concentration were listed in detail (Fig. 3e–g). In DN with macroalbuminuria group, there were positive correlations between serum CN-1 and expressions of FN (*r* = 0.822, *p* = 0.000), Col-IV (*r* = 0.562, *p* = 0.005) and Col-I (*r* = 0.516, *p* = 0.011). In DN with microalbuminuria group, there was a positive correlation between serum CN-1 and tissue expressions of Col-IV (*r* = 0.864, *p* = 0.001). Notably, there was no significant correlation with FN and Col-I in DN with microalbuminuria group. There were no correlations observed between tissue expressions of FN, Col-IV, Col-I and serum CN-1 concentration in MCD group. To test whether tubule damage was involved, we measured the expression of kidney injury molecule-1 (KIM-1) in each group (Fig. 3h, i). According to collected data, more serious tubular injury was found in DN kidney. Moreover, the serum CN-1 concentration and expressions of KIM-1 (*r* = 0.554, *p* = 0.005) showed positive correlations in DN with macroalbuminuria group (Fig. 3j). However, there was no similar correlations found in the other two groups.

### The serum CN-1 expression was not associated with renal inflammation in DN patients

**Fig. 4:**
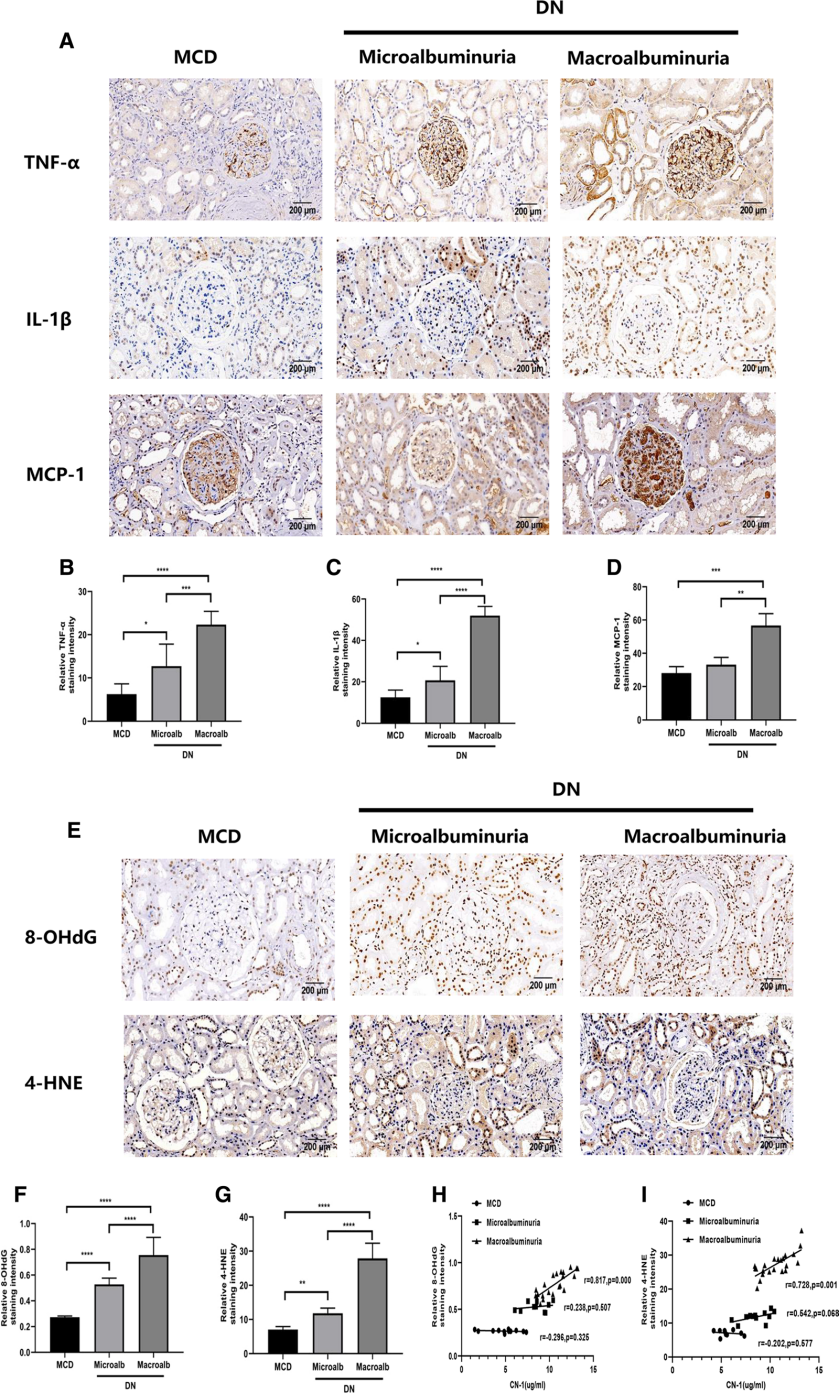
Renal inflammation and oxidative stress correlate with serum CN-1 overexpression in DN with macroalbuminuria. IHC staining of TNF-α, IL-1β and MCP-1 in three groups (**a**). Quantitative results of TNF-α (**b**), IL-1β (**c**) and MCP-1 (**d**). IHC staining of oxidative stress indexes in three groups, including 8-OHdG and 4-HNE (**e**). Quantitative results of 8-OHdG (**f**) and 4-HNE (**g**) in IHC staining. Correlation of serum CN-1 expression with 8-OHdG (**h**) and 4-HNE (**i**). Representative microscopic images are shown (× 200 magnification). *p < 0.05; **p < 0.01; ***p < 0.001; ****p < 0.0001

Renal tissue expressions of tumor necrosis factor-α (TNF-α), monocyte chemoattractant protein-1 (MCP-1) and interleukin-1β (IL-1β) in each group were examined (Fig. 4a–d). Although more sever inflammation was defined in DN kidney, there was no correlation determined between serum CN-1 concentration and renal inflammation.

### The serum CN-1 expression was associated with oxidative stress in DN patient’s kidney

Renal tissue expressions of 8-hydroxy-2′-deoxyguanosine (8-OHdG) and 4-hydroxynonenal (4-HNE) in each group were examined (Fig. 4e–g). Based on collected data, with the increase of urinary protein, the more severe the oxidative stress injury was defined in kidney tissue. Following, we analyzed the correlation between serum CN-1 concentration and renal oxidative stress index (Fig. 4h, i). In DN with macroalbuminuria group, there were positive correlations between serum CN-1 and tissue expressions of 8-OHdG (*r* = 0.817, *p* = 0.000), 4-HNE (*r* = 0.728, *p* = 0.001). In contrast, there is no similar correlations found in MCD group and DN with microalbuminuria group.

## Discussion

First, our results showed that the serum CN-1 concentration and activity in DN group were significantly higher than those in control group, and the serum carnosine content was significantly lower than that in control group. When diabetic nephropathy appears a large amount of proteinuria, its serum CN1 content and activity are increased, especially CN1 activity is at a very high level compared with the other groups. We hypothesized that this was caused by occurrence and progression of diabetic nephropathy. With the prolongation of the disease, the CN-1 contents accumulated in the system and thus caused the high activity of CN-1. This may be related to the enhancement of enzyme activity under long-term hyperglycemia environment. Based on the fact that carnosine concentrations in serum and urine directly depend on the activity of the CN-1, high expression of CN-1 enhanced the carnosine degradation and further suppressed the carnosine protective effects on kidney. This conclusion partially supported the notion that CNDP1 overexpression accelerated the development of diabetes and DN (Qiu et al. 2020). Second, our data also suggested that the serum CN-1 concentration increased during DN development with increasing in albuminuria, serum creatinine, uric acid, and decreasing in serum albumin and eGFR. The results further testified our hypothesis that CN-1 would deteriorate nephropathy by degrading the protective circulating carnosine. However, data presented in this study led to a different conclusion from our previous study on Caucasian T2DM patients, which showed a decreasing trend of serum CN-1 in overt DN (Zhang et al. 2019). The differences might be caused partially by the ethnicity difference as the CTG polymorphism in *CNDP1* gene, which determines the CN-1 secretion efficacy, varies in different population (Janssen et al. 2005; Zhang et al. 2020). Other possible explanation is that the BMI is different between early and overt DN in this study and previous study. Thirdly, the correlation between serum CN-1 concentration and urinary protein components in our study was not only found in glomerular damage biomarkers (i.e., macromolecular Alb, IgG, TRF and micromolecular Cys-C) but also in tubular damage biomarkers (i.e., NAG, RBP, α1-MG and β2-MG), which suggested serum CN-1 could also be a surrogate for DN. To the best of our knowledge, this is the very first study focusing on the association of serum CN-1 with urinary proteins, especially with tubular damages. Although in physiological condition CN-1 locates primarily in distal and proximal tubules in human kidney (Peters et al. 2015), it mainly facilitates DN by enlarging glomerular tuft but not tubules (Qiu et al. 2020), explaining the finding that serum CN-1 was positively correlated to glomerular damage biomarkers. To figure out the relation between serum CN-1 and renal damages, we performed basic pathological staining of biopsy renal tissue in three groups of patients.

In line with the previous study (Peters et al. 2015), our result also found that CN-1 distributed principally in proximal and distal tubules and merely in glomeruli in patients without DN. However, CN-1 relocated as DN progressed; it increased in the glomeruli whereas decreased in the tubules (Fig. 2e). The accumulation of CN-1 in glomeruli in DN patients was also mentioned in Peters’ study. Even so, whether this accumulation was caused by local overexpression of CN-1 or by elevated circulating CN-1 reserved in glomeruli has not yet been determined. Unlike Peters’ study, we found that the CN-1 expression in tubules increased significantly in microalbuminuria patients as compared to MCD patients but slightly decreased in macroalbuminuria patients. This subsequent decrease did not exceed the previous CN-1 expression level in MCD group. As shown in PAS and Masson staining, compared with the MCD group, renal mesangial expansion and tubulointerstitial injury were more sever in DN group. The mesangial expansion and tubule injury were further aggravated as the disease progressed. We hypothesize that CN-1 was excreted from the kidney with massive amounts of proteinuria, resulting in a decrease in CN-1 in the kidney. Previous study has reported the presence of CN-1 in urine (carnosinasuria) in DN patients (Rodriguez‐Niño et al. 2019). Based on this fact, the re-absorption of CN-1might contributes to the escalating CN-1 level in tubules. Although the excessively more severe carnosinasuria was observed in macroalbuminuria patients in the above-mentioned paper, the CN-1 expression was found decreased in tubules from our biopsy staining, which might be as a result of the impaired re-absorptive function in tubules. Nevertheless, further studies on CN-1 overexpression both in vivo and in vitro are needed to clarify the underlying mechanism.

We have further investigated whether renal damage deteriorated in parallel with serum CN-1 concentration. Indeed, the renal fibrosis indicators (i.e., FN, COL-IV, COL-1) (Tessari 2015); the tubular damage indicator (i.e., KIM-1) (Nowak et al. 2016); the inflammatory indicators (i.e., TNF-α, MCP-1 and IL-1β) (Perlman et al. 2015); the oxidative stress indicators (i.e., 8-OHdG and 4-HNE) (Wang et al. 2020) were all increased when albuminuria developed. Moreover, we found that serum CN-1 concentrations were positively associated with the indicators of renal fibrosis, tubular damage, and the oxidative stress in the middle and late stages of DN. The DN with macroalbuminuria group showed the most serious tubular injury and the highest expression KIM-1 renal tissue. This in turn confirmed the accumulation of CN-1 in serum and kidneys were in concomitant with DN progression. Besides, several active carbonyls (RCS), such as 4- HNE, have been shown to be produced under oxidative stress. Moreover, tissue damage and dysfunction are caused by the consumption of glutathione and other reductants to modify proteins, lipids, and DNA. Oxidative stress refers to the increase in the production of reactive oxygen free radicals (ROS) and the decrease in scavenging ability in response to body damage. The reactive oxygen species-induced lipid peroxidation produces a series of RCS, and RCS diffusion amplifies ROS-associated damage (Singh et al. 2015). The excessive or continuous increase of ROS and RCS is related to the onset of diabetes and its complications (Dalle-Donne et al. 2006). Indeed, previous studies also suggested that in addition to the RCS quenching mechanism, the protective effect of carnosine is also partially mediated by free radical scavenging activity and/or transition metal chelation. In addition, carnosine has been proven to inactivate ROS, scavenge free radicals and chelate antioxidant metals (Decker et al. 2000). In fact, carnosine has a particular high affinity for 4-HNE, and reduced levels of carnosine obviously would cause accumulation of HNE-modified proteins. In turn, the high immunogenicity of these protein adducts may cause oxidative stress by receptor-mediated mechanisms, which explain the accumulation of 8-OHdG, an oxidative stress marker of oxidative DNA damage. Higher expression of CN-1 leads to enhanced degradation of carnosine, resulting in decrease in the renal protection of carnosine. Our results showed that the increase in CN-1 levels in DN serum reduced the activity of carnosine in cycling and tissue quenching. This finding is consistent with previous animal experiments (Menini et al. 2012). However, our data showed that although the expressions of inflammatory factors were increased in renal tissue, there were no correlation with serum CN-1 concentration, suggesting that carnosine might mediate the inflammatory response in vivo through other factors. With the verification of the association between CN-1 and renal damage, it is highly possible that CN-1 has some impact at different stages of the DN.

## Conclusion

In this study, we showed that serum CN-1 concentrations increase as urinary proteins increase in DN patients. Moreover, serum CN-1 was associated with clinical and renal injury indicators at different stages of DN. In summary, our studies demonstrated that the expression and activity of CN-1 were increased in DN patients. Serum CN-1 was involved in the development of DN and presented promising therapeutic possibility in the treatment of diabetic nephropathy. Further, our data provided new insights on unveiling the underlying mechanism of diabetic nephropathy. The CN-1 closely related to renal fibrosis, inflammation and oxidative stress in DN kidney. Our data may lay the foundation for a large-scale, in-depth examination of the role of serum CN-1 in DN.

## Data Availability

All data in this paper are derived from published sources and are acknowledged or referenced accordingly.

## References

[CR1] Aldini G, Orioli M, Rossoni G, Savi F, Braidotti P, Vistoli G, Yeum KJ, Negrisoli G, Carini M (2011). The carbonyl scavenger carnosine ameliorates dyslipidaemia and renal function in Zucker obese rats. J Cell Mol Med.

[CR2] American Diabetes Association (2013). Diagnosis and classification of diabetes mellitus. Diabetes Care.

[CR3] An Y, Xu F, Le W, Ge Y, Zhou M, Chen H, Zeng C, Zhang H, Liu Z (2015). Renal histologic changes and the outcome in patients with diabetic nephropathy. Nephrology Dial Transplant.

[CR4] Boldyrev AA, Aldini G, Derave W (2013). Physiology and pathophysiology of carnosine. Physiol Rev.

[CR5] Chakkera HA, Hanson RL, Kobes S, Millis MP, Nelson RG, Knowler WC, Distefano JK (2011). Association of variants in the carnosine peptidase 1 gene (CNDP1) with diabetic nephropathy in American Indians. Mol Genet Metab.

[CR6] Cheng YJ, Kanaya AM, Araneta M, Saydah SH, Kahn HS, Gregg EW, Fujimoto WY, Imperatore G (2019). Prevalence of diabetes by race and ethnicity in the United States, 2011–2016. JAMA.

[CR7] Dalle-Donne I, Aldini G, Carini M, Colombo R, Rossi R, Milzani A (2006). Protein carbonylation, cellular dysfunction, and disease progression. J Cell Mol Med.

[CR8] Decker EA, Livisay SA, Zhou S (2000). A re-evaluation of the antioxidant activity of purified carnosine. Biochemistry.

[CR9] Freedman BI, Hicks PJ, Sale MM, Pierson ED, Langefeld CD, Rich SS, Xu J, McDonough C, Janssen B, Yard BA, van der Woude FJ, Bowden DW (2007). A leucine repeat in the carnosinase gene CNDP1 is associated with diabetic end-stage renal disease in European Americans. Nephrol Dial Transplant.

[CR10] Guariguata L, Whiting DR, Hambleton I, Beagley J, Linnenkamp U, Shaw JE (2014). Global estimates of diabetes prevalence for 2013 and projections for 2035. Diabetes Res Clin Pract.

[CR11] Janssen B, Hohenadel D, Brinkkoetter P, Peters V, Rind N, Fischer C, Rychlik I, Cerna M, Romzova M, de Heer E, Baelde H, Bakker SJ, Zirie M, Rondeau E, Mathieson P, Saleem MA, Meyer J, Köppel H, Sauerhoefer S, Bartram CR, van der Woude FJ (2005). Carnosine as a protective factor in diabetic nephropathy: association with a leucine repeat of the carnosinase gene CNDP1. Diabetes.

[CR12] Koye DN, Magliano DJ, Reid CM, Jepson C, Feldman HI, Herman WH, Shaw JE (2018). Risk of progression of nonalbuminuric CKD to end-stage kidney disease in people with diabetes: the CRIC (Chronic Renal Insufficiency Cohort) Study. Am J Kidney Dis.

[CR13] Krolewski AS (2015). Progressive renal decline: the new paradigm of diabetic nephropathy in type 1 diabetes. Diabetes Care.

[CR14] Lee YT, Hsu CC, Lin MH, Liu KS, Yin MC (2005). Histidine and carnosine delay diabetic deterioration in mice and protect human low density lipoprotein against oxidation and glycation. Eur J Pharmacol.

[CR15] Lenney JF, George RP, Weiss AM, Kucera CM, Chan PW, Rinzler GS (1982). Human serum carnosinase: characterization, distinction from cellular carnosinase, and activation by cadmium. Clin Chim Acta Int J Clin Chem.

[CR16] Menini S, Iacobini C, Ricci C, Scipioni A, Blasetti Fantauzzi C, Giaccari A, Salomone E, Canevotti R, Lapolla A, Orioli M, Aldini G, Pugliese G (2012). d-Carnosine octylester attenuates atherosclerosis and renal disease in ApoE null mice fed a Western diet through reduction of carbonyl stress and inflammation. Br J Pharmacol.

[CR17] Nowak N, Skupien J, Niewczas MA, Yamanouchi M, Major M, Croall S, Smiles A, Warram JH, Bonventre JV, Krolewski AS (2016). Increased plasma kidney injury molecule-1 suggests early progressive renal decline in non-proteinuric patients with type 1 diabetes. Kidney Int.

[CR18] Pegova A, Abe H, Boldyrev A (2000). Hydrolysis of carnosine and related compounds by mammalian carnosinases. Comp Biochem Physiol Part B Biochem Mol Biol.

[CR19] Perkins BA, Ficociello LH, Ostrander BE, Silva KH, Weinberg J, Warram JH, Krolewski AS (2007). Microalbuminuria and the risk for early progressive renal function decline in type 1 diabetes. J Am Soc Nephrol.

[CR20] Perlman AS, Chevalier JM, Wilkinson P, Liu H, Parker T, Levine DM, Sloan BJ, Gong A, Sherman R, Farrell FX (2015). Serum inflammatory and immune mediators are elevated in early stage diabetic nephropathy. Ann Clin Lab Sci.

[CR21] Peters V, Kebbewar M, Jansen EW, Jakobs C, Riedl E, Koeppel H, Frey D, Adelmann K, Klingbeil K, Mack M, Hoffmann GF, Janssen B, Zschocke J, Yard BA (2010). Relevance of allosteric conformations and homocarnosine concentration on carnosinase activity. Amino Acids.

[CR22] Peters V, Klessens CQ, Baelde HJ, Singler B, Veraar KA, Zutinic A, Drozak J, Zschocke J, Schmitt CP, de Heer E (2015). Intrinsic carnosine metabolism in the human kidney. Amino Acids.

[CR23] Qiu J, Albrecht T, Zhang S, Hauske SJ, Rodriguez-Niño A, Zhang X, Nosan D, Pastene DO, Sticht C, Delatorre C, van Goor H, Porubsky S, Krämer BK, Yard BA (2020). Human carnosinase 1 overexpression aggravates diabetes and renal impairment in BTBR^Ob/Ob^ mice. J Mol Med (Berl).

[CR24] Riedl E, Koeppel H, Brinkkoetter P, Sternik P, Steinbeisser H, Sauerhoefer S, Janssen B, van der Woude FJ, Yard BA (2007). A CTG polymorphism in the CNDP1 gene determines the secretion of serum carnosinase in Cos-7 transfected cells. Diabetes.

[CR25] Robles NR, Villa J, Gallego RH (2015). Non-proteinuric diabetic nephropathy. J Clin Med.

[CR26] Rodriguez-Niño A, Gant CM, Braun JD, Li X, Zhang S, Albrecht T, Qiu J, Bakker S, Laverman GD, Krämer BK, Herold A, Hauske SJ, Yard BA (2019). Detection of carnosinase-1 in urine of healthy individuals and patients with type 2 diabetes: correlation with albuminuria and renal function. Amino Acids.

[CR27] Sauerhöfer S, Yuan G, Braun GS, Deinzer M, Neumaier M, Gretz N, Floege J, Kriz W, van der Woude F, Moeller MJ (2007). l-Carnosine, a substrate of carnosinase-1, influences glucose metabolism. Diabetes.

[CR28] Singh M, Kapoor A, Bhatnagar A (2015). Oxidative and reductive metabolism of lipid-peroxidation derived carbonyls. Chem Biol Interact.

[CR29] Tessari P (2015). Nitric oxide in the normal kidney and in patients with diabetic nephropathy. J Nephrol.

[CR30] Teufel M, Saudek V, Ledig JP, Bernhardt A, Boularand S, Carreau A, Cairns NJ, Carter C, Cowley DJ, Duverger D, Ganzhorn AJ, Guenet C, Heintzelmann B, Laucher V, Sauvage C, Smirnova T (2003). Sequence identification and characterization of human carnosinase and a closely related non-specific dipeptidase. J Biol Chem.

[CR31] Wang D, Li Y, Wang N, Luo G, Wang J, Luo C, Yu W, Hao L (2020). 1α,25-Dihydroxyvitamin D_3_ prevents renal oxidative damage via the PARP1/SIRT1/NOX4 pathway in Zucker diabetic fatty rats. Am J Physiol Endocrinol Metab.

[CR32] Whyte MB, Hinton W, McGovern A, van Vlymen J, Ferreira F, Calderara S, Mount J, Munro N, de Lusignan S (2019). Disparities in glycaemic control, monitoring, and treatment of type 2 diabetes in England: a retrospective cohort analysis. PLoS Med.

[CR33] Zhang S, Albrecht T, Rodriguez-Niño A, Qiu J, Schnuelle P, Peters V, Schmitt CP, van den Born J, Bakker S, Lammert A, Krämer BK, Yard BA, Hauske SJ (2019). Carnosinase concentration, activity, and CNDP1 genotype in patients with type 2 diabetes with and without nephropathy. Amino Acids.

[CR34] Zhang S, Xu J, Cui D, Jiang S, Xu X, Zhang Y, Zhu D, Xia L, Yard B, Wu Y, Zhang Q (2020). Genotype distribution of *CNDP1* polymorphisms in the healthy Chinese Han population: association with HbA1c and fasting blood glucose. J Diabetes Res.

